# Design of Howland Current Sources Using Differential Evolution Optimization

**DOI:** 10.2478/joeb-2020-0014

**Published:** 2020-12-31

**Authors:** Kaue Felipe Morcelles, Lucas Hermann Negri, Pedro Bertemes-Filho

**Affiliations:** 1 State University of Santa Catarina. Joinville. Brazil; 2 Federal Institute of Education, Science and Technology of Mato Grosso do Sul. Nova Andradina, Brazil.; 3 State University of Santa Catarina. Joinville, Brazil

**Keywords:** bioimpedance, differential evolution, Howland source, optimization

## Abstract

Howland circuits have been widely used in Electrical Bioimpedance Spectroscopy applications as reliable current sources. This paper presents an algorithm based on Differential Evolution for the automated design of Enhanced Howland Sources according to arbitrary design constraints while respecting the Howland ratio condition. Results showed that the algorithm can obtain solutions to commonly sought objectives, such as maximizing the output impedance at a given frequency, making it a versatile method to be employed in the design of sources with specific requirements. The mathematical modeling of the source output impedance and transconductance, considering a non-ideal operational amplifier, was validated against SPICE simulations, with results matching up to 10 MHz.

## Introduction

Electrical Bioimpedance Spectroscopy (EBS) has been widely used over the last 50 years for biomedical applications [1], such as cancer diagnoses [2], monitoring of physiological activity (lung fluids [3], muscle contractions [4] and cardiac output [5]) and real-time tomography [6]. EBS uses the correlation between material constitution/geometry and electrical impedance to characterize the tissue [7].

The Enhanced Howland Source (EHS) is a current pump used in EBS measurements consisting of one operational amplifier and five resistors [8]. Its parameters (transconductance, output impedance, voltage compliance, noise) can be designed by selecting adequate resistor values and OPAMP model [9, 8, 7]. The transconductance value and minimum voltage compliance are usually fixed by the application requirements, whereas the output impedance and noise should be maximized and minimized, respectively, to avoid measurement errors. Especially concerning the output impedance (Zout), the EHS has limitations in high frequencies.

This paper proposes the usage of differential evolution to design a Howland current source with optimal output impedance according to arbitrary project constraints.

## Methodology

The Differential Evolution (DE) optimization method [10] was employed to select the optimal resistor values (*R*_1_, *R*_2__*A*_, *R*_2__*B*_, *R*_3_, and *R*_4_) for the Howland Source, according to the design goals (see Figure 2).

**Figure 1:**
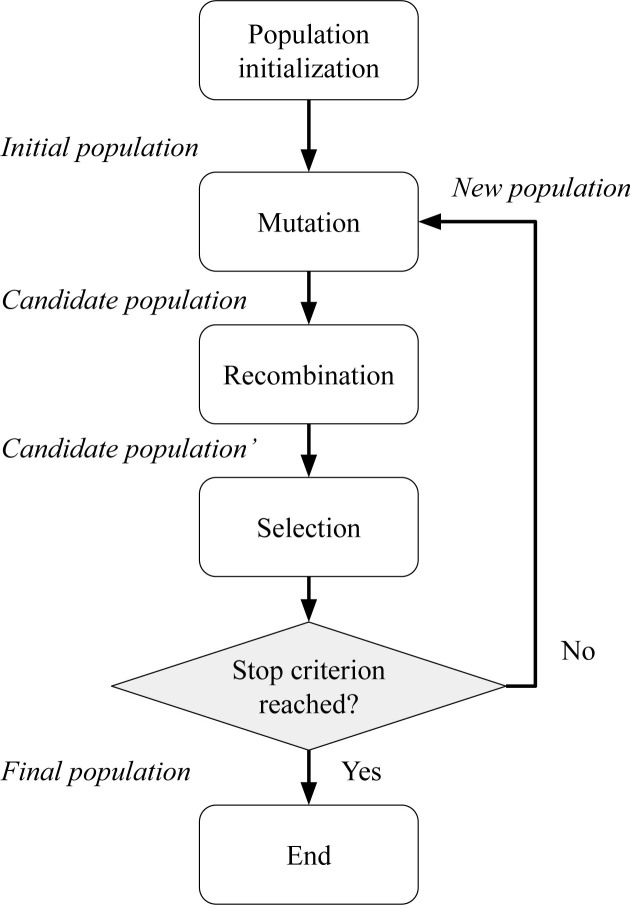
Flowchart that describes the main steps of the optimization method.

Being a black-box, population-based metaheuristic, the DE does not use gradient information to guide the optimization process. Instead, DE relies in a fitness function, that determines the quality of a candidate solution. Specifically, the JADE variant of DE was employed [11] to make the optimization process more robust, as JADE automatically updates the DE control parameters. Figure 1 shows a flowchart that describes the general steps of the optimization method.

The optimization procedure is based on the mathematical modeling of the Howland Source, and was performed to maximize the output impedance at the highest frequency of operation. A total of 50 candidate solutions were evolved during 1000 iterations of the optimization procedure.

The mathematical model of the Howland Source used in this work includes the resistor set (*R*_1_, *R*_2__*A*_, *R*_2__*B*_, *R*_3_, *R*_4_) and the Operational Amplifier (OPAMP) non-idealities that potentially affects the parameters of interest, namely the finite open-loop gain *A*, finite cut-off frequency *f*_*c*_, common mode input resistance *R*_*CM*_. As the algorithm can yield large values of resistors, the input capacitance of the OPAMP (*C*_*in*_) must also be included on the model, in order to predict instability and high frequency peaks due to phase margin reduction at the negative feedback loop.

**Figure 2:**
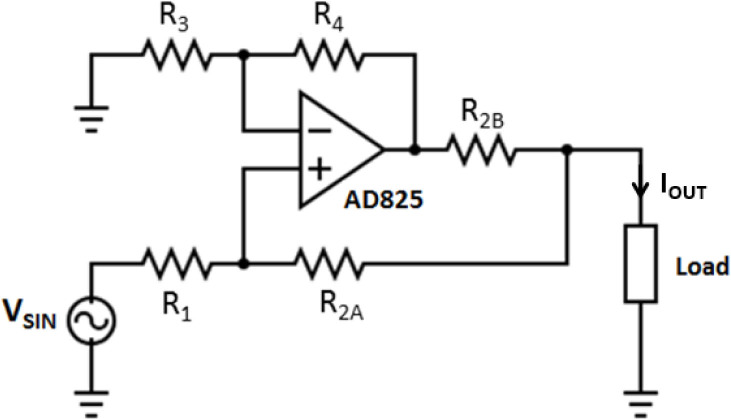
Schematic circuit of the ground loaded modified Howland Current Source.

The output impedance and transconductance were calculated using Equations 1 and 2. Maximum load capability was evaluated using the model presented by a previous work [12]. The equations were derived from the Howland circuit shown in Figure 2.

(1) $$Z_{\mathrm{out}}=\frac{R_{2A}\left(s+\frac{1}{2\;C_{\mathrm{in}}\left(R_{\mathrm{CM}}\left\|R_{1}\right\|R_{2A}\right)}\right)}{-\frac{A\left(s+\frac{1}{2\;C_{\mathrm{in}}\left(R_{\mathrm{CM}}\left\|R_{3}\right\|R_{4}\right)}\right)}{R_{2B}\left(\left(2\;C_{\mathrm{in}}s+\frac{1}{\left(R_{\mathrm{CM}}\left\|R_{3}\right\|R_{4}\right)}\right)\left(\frac{s}{f_{c}\pi }+1\right)\frac{A}{R_{4}}\right)}+\left(\frac{R_{2A}}{R_{2B}}+1\right)\left(s+\frac{1}{2\;C_{\mathrm{in}}\left(R_{\mathrm{CM}}\left\|R_{1}\right\|R_{2A}\right)}\right)-\frac{1}{2\;C_{\mathrm{in}}R_{2A}}}$$

(2) $$G=\frac{AR_{4}R_{\mathrm{CM}}}{\left(\frac{s}{2\;f_{c}\pi }+1\right)\left(R_{\mathrm{CM}}\left(R_{2B}R_{3}\left(\frac{A}{\frac{s}{2\;f_{c}\pi }+1}+1\right)+R_{2B}R_{4}\right)+C_{\mathrm{in}}R_{2B}R_{3}R_{4}R_{\mathrm{CM}}s+R_{2B}R_{3}R_{4}\right)}$$

The fitness function employed in the selection step of the JADE method is given by equation 3, where *p*_1_, *p*_2_, *p*_3_, and *p*_4_ (Equations 4, 5, 6, 7) are penalty factors that implements the design constraints, *α* is the penalization weight that is calibrated (increased until all design constraints are met), *rl*_*max*_ is given by equation 8, and *G*_*sample*_ is a vector with samples of the transconductance magnitude in the 100 kHz to 1 MHz band. For the Optimized Case 1, the transconductance variance was not taken into account, therefore *β* = 0 at band. For the Optimized Case 1, the transconductance equation 6. For the Optimized Case 2, the value *β* = 10^−9^ was used instead.

(3) $$\mathrm{fitness}=Z_{\mathrm{out}}-\alpha \left(p_{1}+p_{2}+p_{3}+p_{4}\right)$$

(4) $$p_{1}=\left|\frac{R_{4}}{R_{3}R_{2b}G_{p}}-1\right|$$

(5) $$p_{2}=\left|\frac{\left(R_{2a}+R_{2b}\right)R_{3}}{R_{1}R_{4}}-1\right|$$

(6) $$p_{3}=\beta \frac{\mathrm{var}\left(G_{\mathrm{sample}}\right)}{\mathrm{median}\left(G_{\mathrm{sample}}\right)}$$

(7) $$p_{4}=1-\frac{rl_{\text{max}}}{Rl_{\text{max}}}$$

(8) $$rl_{\text{max}}=\frac{V_{\mathrm{sat}}}{V_{\mathrm{in}}G_{p}}{\left(1+\frac{R_{2B}}{R_{2A}+R_{1}}\right)}^{-1}$$

### Ethical approval

The conducted research is not related to either human or animal use.

## Results

**Figure 3:**
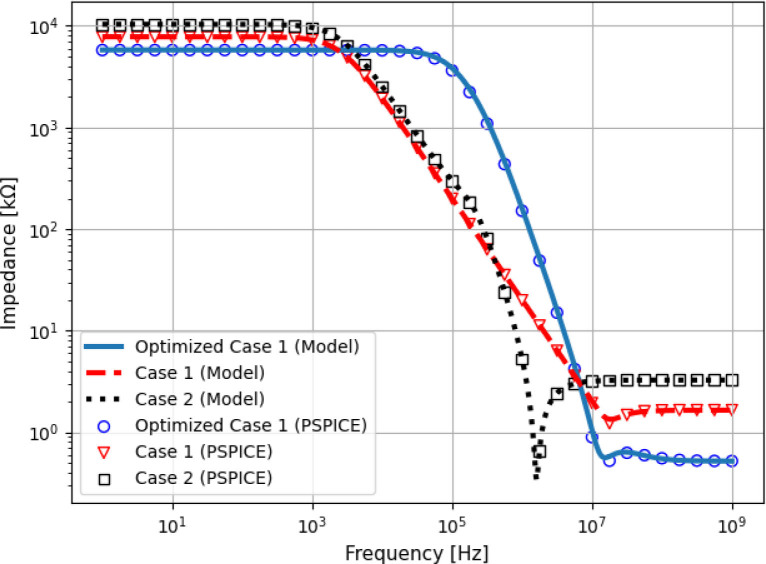
Output impedance of the optimized and non-optimized Howland Source circuits.

**Figure 4:**
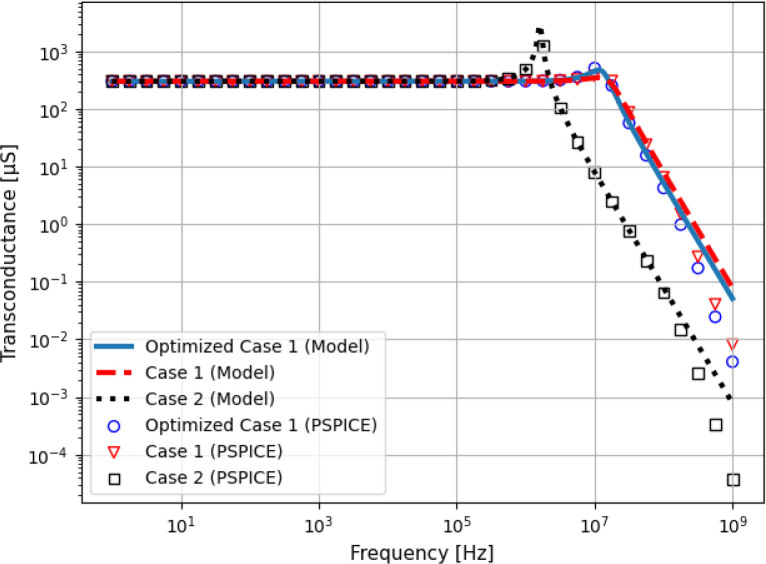
Transconductance curve of the optimized and non-optimized Howland Source Circuit.

In order to validate the algorithm, a case study was performed, consisting of the design of a Howland circuit with a set of pre-defined requirements (Table 1). The operational amplifier model used was the AD825, from Analog Devices™. The algorithm was configured to automatically design the resistor values of the Howland Source, maximizing the output impedance modulus at the maximum desired frequency (in this case 1.2 MHz), while respecting the design requirements and the Howland ratio condition [9]. Also, the resistors were constrained to values between 0.05 and 100 *k*Ω. The first round of optimization evaluated a solution (Optimized Case 1) consisting of *R*_1_ = 4.303990 *k*Ω, *R*_2__*A*_ = 0.678260 *k*Ω, *R*_2__*B*_ = 2.230320 *k*Ω, *R*_3_ = 7.478330 *k*Ω and *R*_4_ = 5.053760 *k*Ω. The resulted impedance and transconductance curves are compared with two cases: Case 1 consisted of the common approach of making *R*_2__*B*_ = *R*_2__*A*_ = *R*_3_ = *R*_4_ = *R*_1_/2 and *G* = 1/*R*_2__*B*_, a combination that is not yet optimized for output impedance [12]. In this case, the requirements of Table 1 were satisfied using *R*_2__*B*_ = 3.3 *k*Ω [12]. Case 2 verified the impact of increasing the feedback resistors, making *G* = 1/*R*_2__*B*_, *R*_2__*A*_ = *R*_3_ = *R*_4_ = 100*R*_2__*B*_ and *R*_1_ = *R*_2__*A*_ + *R*_2__*B*_. This approach reduces the feedback current, increasing voltage swing. In this case, *R*_2__*B*_ = 3.3 *k*Ω was also used. The resulted output impedance and transconductance (no load) are shown in Figures 3 and 4.

**Table 1:** Electrical parameters required for designing the Howland current source.

Transconductance (*G*_*p*_)	303.03 *μS*
Frequency Range	10 Hz - 1.2 MHz
Maximum Load (*Rl*_*max*_)	2.0 kΩ
Supply Voltage (*V*_*sat*_)	± 5.0 V
Input Voltage (*V*_*in*_)	1.65 V
Operational Amplifier	AD825

At maximum frequency (1.2 MHz), Case 1 and Case 2 obtained 16.80 *k*Ω and 2.85 *k*Ω respectively, whereas the Optimized Case 1 obtained 107.61 *k*Ω, which is 6.4 times higher than Case 1. In contrast, increasing the feedback resistors (Case 2) incremented the output impedance only at low frequencies, leading to a reduction in *Z*_*out*_ at high frequencies due to interactions with the input capacitance of the operational amplifier. All cases presented transconductance peaking at high frequencies, but due to the strong effects of the input capacitance Case 2 presented the largest current error. Finally, it can be seen that the curves generated with PSPICE and the ones evaluated using the equations here presented agree up to 10 MHz.

**Figure 5:**
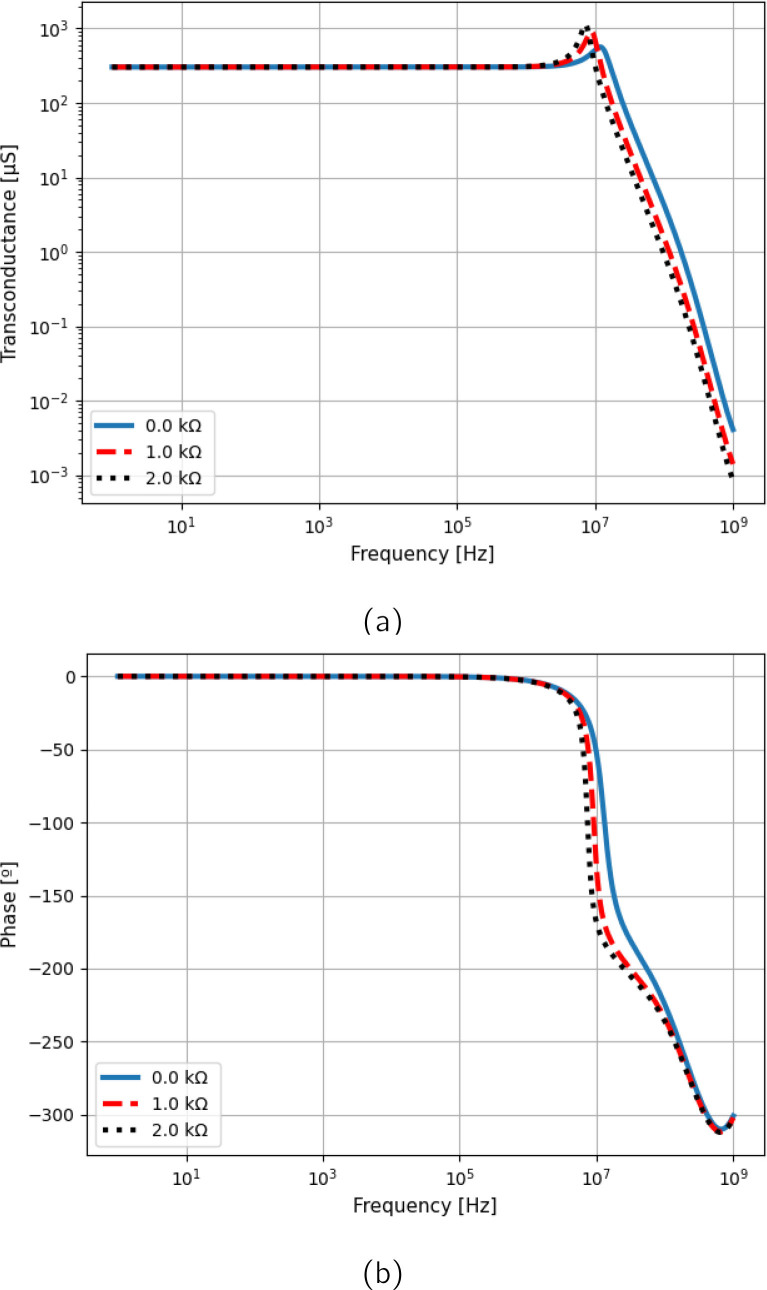
Transconductance (a) magnitude and (b) phase curves of the Optimized Case 1 for three different loads.

**Figure 6:**
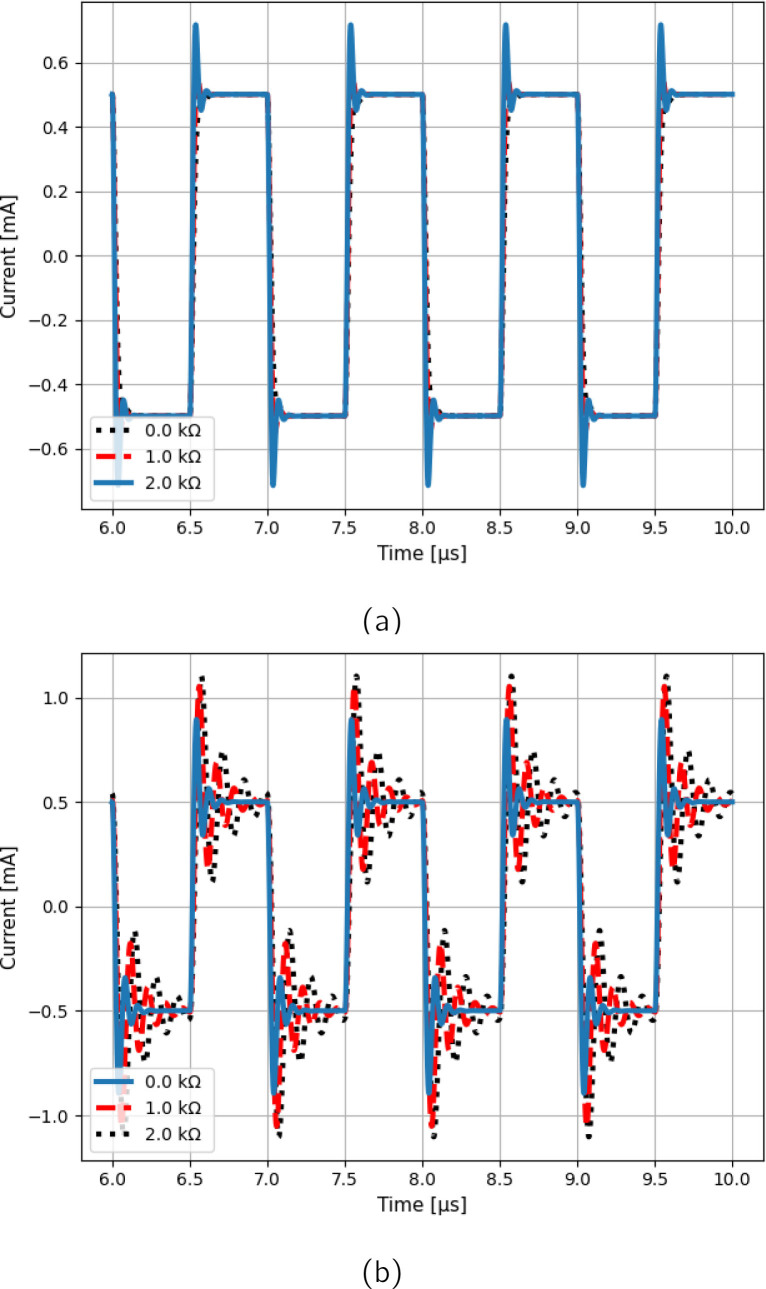
Output current of the (a) Case 1 and (b) Optimized Case 1 circuits for three different loads and 1 MHz square wave input voltage.

To verify the dependence of the output current on the load, Figure 5 shows the transconductance of the Optimized Case 1 curves for three different loads. It can be seen that the high frequency current peaking increases with the load, which is the opposite of what happens in Case 1. To verify the time domain effects of peaking, Figure 6 shows the wave-shape of the output current of Case 1 and the Optimized Case 1, considering three different loads. The Optimized Case 1 presented a higher overshoot value that increased with the load, whereas Case 1 obtained lower overshoot that decreased with the load. This behavior may be problematic in sensitive applications measuring high loads, as high overshoot degrades signal performance and resolution, especially when using square waves or other discrete signals. Peaking can be reduced by placing a capacitor in parallel with *R*_4_, but this has the cost of significantly reducing the output impedance at high frequencies.

To minimize the current peaking, another run of optimization was performed, generating the resistor set called Optimized Case 2, using *β* = 10^−9^ at equation 6. This approach maximizes the output impedance while lowering the amount of peaking. The algorithm evaluated the solution: *R*_1_ = 4.187160 *k*Ω, *R*_2__*A*_ = 1.192640 *k*Ω, *R*_2__*B*_ = 4.438410 *k*Ω, *R*_3_ = 2.529140 *k*Ω and *R*_4_ = 3.401290 *k*Ω. The curve had a peak of 6.20% (in contrast with Optimized Case 1, with peak of 57.22%). However, the *Z*_*out*_ decreased to the level of Case 1, being slightly higher (20 *k*Ω) at maximum frequency. This result illustrates a trade-off between output impedance and high frequency peaking presented in the circuits generated by the algorithm, and the designer should decide which parameter is more important for the application.

Although the Optimized Case 1 presented higher current peaking than the Case 1 and the Optimized Case 2, the errors within the bandwidth of interest (up to 1.2 MHz) were significantly smaller, as shown in Tables 2 and 3. In the Optimized Case 1, both magnitude and phase errors were smaller even at maximum load (2.0 *k*Ω), which is the case of maximum peaking for the Optimized Case 1. This result was expected, as higher output impedance typically results in lower output current variations with the load.

**Table 2:** Output Current Error (Magnitude and Phase) for different Loads (at 100 kHz).

Case	Load [*k*Ω]	Magnitude [%]	Phase [°]
Case 1	0.0	0.62	−0.36
	1.0	1.13	−0.65
	2.0	1.63	−0.93

Optimized 1	0.0	0.52	−0.30
	1.0	0.55	−0.31
	2.0	0.57	−0.33

Optimized 2	0.0	0.56	−0.32
	1.0	0.99	−0.57
	2.0	1.42	−0.81

**Table 3:** Output Current Error (Magnitude and Phase) for different Loads (at 1.2 MHz).

Case	Load [*k*Ω]	Magnitude [%]	Phase [°]
Case 1	0.0	7.55	−4.32
	1.0	13.50	−7.75
	2.0	19.33	−11.14

Optimized 1	0.0	6.39	−3.67
	1.0	6.91	−3.82
	2.0	7.53	−4.01

Optimized 2	0.0	6.78	−3.87
	1.0	11.95	−6.84
	2.0	17.10	−9.81

Although the JADE algorithm is capable of finding the resistor combination that yields maximum output impedance, it does not take into account the variations due to fabrication tolerances. Therefore, Monte Carlo simulations were performed in PSPICE to evaluate the robustness of the circuit solution. In this test, precision resistors with 0.05% tolerances were considered. The Monte Carlo simulation ran 10000 cases (using Gaussian distribution), checking the variation of the output impedance value at the maximum frequency (1.2 MHz). Results are shown in Table 4, with the mean (*μ*), standard deviation (*σ*) and coefficient of variation (CV) of the output impedance of each case. It can be seen from Table 4 that the solutions provided by the JADE algorithm are less robust than Case 1, but still provide a significant increase in output impedance at the desired frequency. Better robustness can be obtained using resistors with lower tolerances.

**Table 4:** Monte Carlo simulation results (at 1.2 MHz), showing the mean output impedance (*μ*), standard deviation (*σ*) and the coefficient of variation (CV) of each case over 10000 runs using 0.05% tolerance resistors.

Case	*μ* [*k*Ω]	*σ* [*k*Ω]	CV [%]
Case 1	16.26	1.18	7.25
Optimized	103.41	29.89	28.90
Optimized 2	18.67	2.05	10.96

## Conclusion

The paper investigated the use of differential evolution for designing and modelling a Howland current source with optimum output impedance and complying with project constraints. It was focused on the output impedance optimization at 1.2 MHz, but can be extrapolated to different frequencies and parameters. It was found a trade off between the high frequency transconductance error and output impedance during the optimization process. Decreasing the transconductance peak can be done by minimizing transconductance variance, however at price of having a decrease in the output impedance. Nevertheless, it was showed that the mathematical model and simulations agreed significantly up to 10 MHz. The analytical nature of the models make them more suitable for iterative optimization algorithms, providing less computational cost and time when compared with numerical and SPICE based optimization approaches. This is a promising output to be used in the design of bioimpedance measurement circuits which, in turns, are very dependent on the clinical application and the biological samples under study. Future work will focus on extracting experimental data, to verify how the algorithm complies with parasitic stray capacitances, non-idealities of the components and the limited availability of discrete valued resistors.
